# CCDC88A mutations cause PEHO-like syndrome in humans and mouse

**DOI:** 10.1093/brain/aww014

**Published:** 2016-02-25

**Authors:** Michael S. Nahorski, Masato Asai, Emma Wakeling, Alasdair Parker, Naoya Asai, Natalie Canham, Susan E. Holder, Ya-Chun Chen, Joshua Dyer, Angela F. Brady, Masahide Takahashi, C. Geoffrey Woods

**Affiliations:** ^1^ Cambridge Institute for Medical Research, University of Cambridge, Cambridge, CB2 0XY, UK; ^2^ Department of Pathology, Centre for Neurological Disease and Cancer, Nagoya University Graduate School of Medicine, 65 Tsurumai-cho, Showa-ku, Nagoya, Aichi, 466N, Japan; ^3^ North West Thames Regional Genetics Service, Level 8V, London North West Healthcare NHS Trust, Watford Road, Harrow, HA1 3UJ, UK; ^4^ Department of Paediatric Neuroscience, Addenbrooke’s Hospital, Hills Rd, Cambridge, CB2 0QQ, UK

**Keywords:** PEHO syndrome, girdin, neurodevelopmental disorder, epilepsy, microcephaly

## Abstract

Progressive encephalopathy with oedema, hypsarrhythmia and optic atrophy (PEHO) syndrome is a rare Mendelian phenotype comprising severe retardation, early onset epileptic seizures, optic nerve/cerebellar atrophy, pedal oedema, and early death. Atypical cases are often known as PEHO-like, and there is an overlap with ‘early infantile epileptic encephalopathy’. PEHO is considered to be recessive, but surprisingly since initial description in 1991, no causative recessive gene(s) have been described. Hence, we report a multiplex consanguineous family with the PEHO phenotype where affected individuals had a homozygous frame-shift deletion in *CCDC88A* (c.2313delT, p.Leu772*ter). Analysis of cDNA extracted from patient lymphocytes unexpectedly failed to show non-sense mediated decay, and we demonstrate that the mutation produces a truncated protein lacking the crucial C-terminal half of CCDC88A (girdin). To further investigate the possible role of *CCDC88A* in human neurodevelopment we re-examined the behaviour and neuroanatomy of *Ccdc88a* knockout pups. These mice had mesial-temporal lobe epilepsy, microcephaly and corpus callosum deficiency, and by postnatal Day 21, microcephaly; the mice died at an early age. As the mouse knockout phenotype mimics the human PEHO phenotype this suggests that loss of CCDC88A is a cause of the PEHO phenotype, and that CCDC88A is essential for multiple aspects of normal human neurodevelopment.

## Teaser

P rogressive E ncephalopathy with oedema, H ypsarrhythmia and O ptic atrophy (PEHO) is a rare, neurodegenerative disorder of unknown aetiology. Nahorski *et al* . identify the first causative recessive mutation in *CCDC88A* , which encodes the actin-binding protein Girdin. The phenotype and brain anatomy of the *Ccdc88a* knockout mouse resemble those of human PEHO syndrome.

## Graphical abstract

**Figure:**
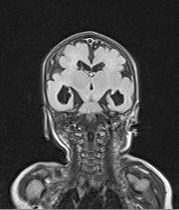
P rogressive E ncephalopathy with oedema, H ypsarrhythmia and O ptic atrophy (PEHO) is a rare, neurodegenerative disorder of unknown aetiology. Nahorski *et al* . identify the first causative recessive mutation in *CCDC88A* , which encodes the actin-binding protein Girdin. The phenotype and brain anatomy of the *Ccdc88a* knockout mouse resemble those of human PEHO syndrome.

## Introduction

Progressive encephalopathy with oedema, hypsarrhythmia and optic atrophy (PEHO) syndrome is rare, of unknown origin, and causes profound intellectual disability with little if any developmental progress. It was first described in Finnish children in 1991, from where the most complete phenotypic data originates ( Salonen *et al.* , 1991 ). Onset occurs usually within the first few weeks of life with affected children displaying seizures, hypotonia and lack of visual fixation ( Somer, 1993 ; D'Arrigo *et al.* , 2005 ). Those affected thence exhibit early arrest of motor and mental development, optic atrophy, with radiological examination demonstrating severe cerebellar and brainstem atrophy from birth, with cerebral atrophy developing at a later stage ( D'Arrigo *et al.* , 2005 ). Usually microcephaly is present at birth, but inevitably there is a progressive reduction in brain growth (secondary microcephaly). Prognosis is poor with most patients dying before 10 years of age. Inheritance is predicted to be autosomal recessive ( Somer, 1993 ). Clinically there is phenotypic overlap of PEHO syndrome with the early-infantile epileptic encephalopathies ( Cross and Guerrini, 2013 ). Cases lacking either optic atrophy or cerebellar hypoplasia are often termed PEHO-like ( Chitty *et al.* , 1996 ).

The genetic cause(s) and the underlying pathophysiology of PEHO syndrome are unknown; and there is no known curative therapy. We report a large consanguineous family with three affected members in two nuclear families. In affected individuals we identified a homozygous frameshift mutation in *CCDC88A* , the gene that encodes the actin binding protein girdin (GIRDers of actIN filament, also known as APE GIV and HkPP) ( Anai *et al.* , 2005 ; Enomoto *et al.* , 2005 ; Le-Niculescu *et al.* , 2005 ; Simpson *et al.* , 2005 ). We show that the mutation results in the absence of full length, functional CCDC88A protein. To further investigate the function of CCDC88A in mammalian brain function we examined CCDC88A-deficient mice and compare their phenotype and CNS architecture to our patients. Our results reveal novel insights into the underlying developmental, neuroanatomical and molecular defects of CCDC88A deficiency in mammals.

## Materials and methods

Materials and methods are described in detail in the Supplementary material .

### Subjects

The UK National Research Ethics Service, Cambridge Central Research Ethics committee gave permission for our study. The authors cared for the family for over a decade; all medical care was within the National Health Service. Results and notes were examined and recorded.

### Exome sequencing

Genomic DNA from affected Patients 1 and 2 ( Fig. 2 A) was extracted and analysed by exome sequencing (SureSelect Human All Exon 50Mb Kit, Agilent Technologies). Potentially pathogenic mutations were identified by comparison with the GRCh37 reference human genome, filtering for changes with allele frequencies of < 1 in 500 in the 1000 Genomes project, and focused on homozygous changes affecting both siblings. Any changes were confirmed and tested for expected segregation, by polymerase chain reaction (PCR) and Sanger sequencing.

**Figure 1:**
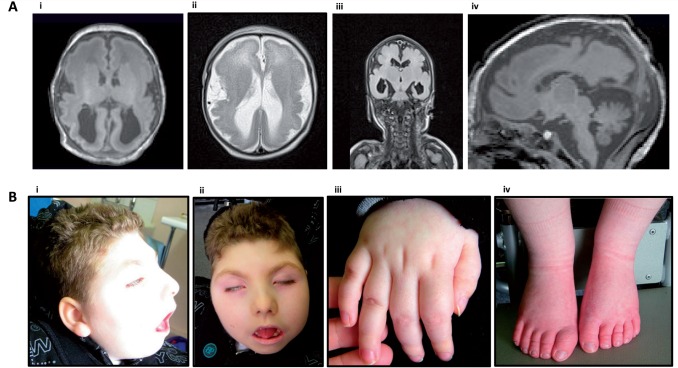
**
Clinical features of PEHO syndrome caused by *CCDC88A* truncating mutation.** ( **A** ) Brain scans of affected patients. [ **A** ( **i** and **ii** )] T _1_ - and T _2_ -weighted axial images; [ **A(iii)** ] T _1_ SENSE coronal images. All demonstrate reduced brain volume with bilateral, severe pachygyria/lissencephaly. [ **A** ( **iv** )] represents a T _2_ -weighted TIRM sagittal image demonstrating a thin corpus callosum and mild reduction in cerebellar vermis volume. [ **B** ( **i** and **ii** )] Characteristic faces with apparent swollen cheeks, narrow sloping forehead and microcephaly. [ **B** ( **iii** )] Typical oedema of the dorsum of the hand. [ **B** ( **iv** )] Typical pedal oedema of PEHO syndrome. Images are of the proband Patient 1 at age 6 years.

**Figure 2:**
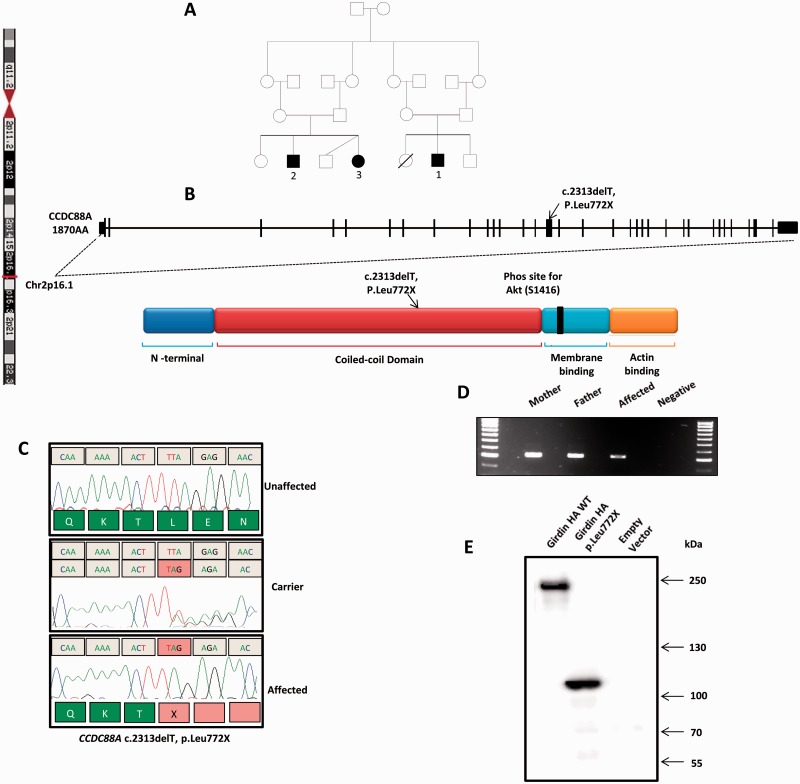
**
Discovery of a c.2313delT/p.Leu772X mutation in *CCDC88A* in individuals with PEHO syndrome.** ( **A** ) Family pedigree with both sets of parents being first cousins. The filled in symbols indicate the three affected children; ‘1’ was the proband. ( **B** ) Schematic diagram of the *CCDC88A* gene on chromosome 2, and girdin protein with its known functional domains annotated. The c.2313delT mutation occurs in exon 15 and causes a nonsense mutation producing a truncated protein product lacking the membrane binding and actin binding domains of girdin. ( **C** ) Electrophoretograms of an unaffected control, carrier parent and affected child with the p.Leu772X mutation. ( **D** ) Reverse transcriptase polymerase chain reaction amplifying the mutant cDNA transcript from mRNA extracted from the lymphocytes of affected Patient 1 and his parents. ( **E** ) Western blot probed for haemagglutinin (HA), demonstrating the truncated product of *CCDC88A* harbouring the c.2313delT mutation.

### Reverse transcription polymerase chain reaction analysis

To analyse possible nonsense mediated decay of mutant CCDC88A, blood samples were taken from Patient 1 and their parents, mRNA extracted and reverse transcribed to cDNA. Full details of reverse transcription PCRs (RT-PCRs) undertaken and primers used are detailed in the Supplementary material .

### *CCDC88A* cloning and expression

*CCDC88A* cloned into the pCAGGS vector has been described previously ( Enomoto *et al.* , 2005 ). The c.2313delT mutation was introduced into the wild-type *CCDC88A* sequence by site-directed mutagenesis using the QuikChange II Site-Directed Mutagenesis kit (Agilent), and a haemagglutinin (HA) tag added to the N-terminus. Expression of wild-type and mutant proteins were analysed by western blot.

### *CCDC88A* knockout mouse

The *CCDC88A* knockout mice and conditional Nestin–cre knockout mice strains have been reported previously ( Kitamura *et al.* , 2008 ; Asai *et al.* , 2012 ). Full details of brain dissection, staining and analysis are described in the Supplementary material .

## Results

### Clinical features of the family

The clinical features of the affected children are summarized in Table 1 , and individual histories are given in the Supplementary material .

**Table 1:** **
Diagnostic criteria for PEHO syndrome, with our clinical data and that of the *Ccdc88a* knockout mice
**

**Clinical criteria for PEHO**	**Patient** 1	**Patient 2**	**Patient 3**	***Ccdc88a* knockout mouse **
**Necessary criteria**				
Infantile, usually neonatal hypotonia	Yes	Yes	Yes	Mice underweight ( Asai *et al.* , 2012 ) Postnatal lethality ( Asai *et al.* , 2012 )
Profound psychomotor retardation with severe hypotonia: absence of motor milestones and speech	Yes	Yes	Yes	Not tested; however, we note possible behavioural differences: *Ccdc88a* mice do not hang upside down from the cage like normal mice.
				‘Mice were inactive, and exhibited growth retardation and fatality after P12’ ( Asai *et al.* , 2012 )
Convulsive disorders presenting with myoclonic jerking and infantile spasms	Yes	Yes	Yes	Mesial-temporal lobe epilepsy phenotype ( Asai *et al.* , 2012 )
Absence or early loss of visual fixation with atrophy of optic disc by 2 years of age	Yes	Yes	Yes	No
Progressive brain atrophy in neuroimaging studies. Particularly affecting the cerebellum and brain stem; milder supratentorial atrophy	Yes	Yes	Yes	Developmental defect in caudal end of corpus callosum Small cerebrum at postnatal Day 0
**Supportive criteria**				
Subtle dysmorphic features including narrow forehead, epicanthic folds, short nose, open mouth, receding chin and tapering fingers	Yes	Yes	Yes	N/A
Oedema of the face and limbs (early childhood)	Yes	Yes	Yes	Not identified
Brisk tendon reflexes (early childhood)	Yes	Yes	Yes	N/A
Absent cortical responses of somatorysensory evoked potentials	Yes	Not done	Yes	N/A
Slow nerve conduction velocities in late childhood	Not done	Not done	Not done	N/A
Dysmyelination in MRI	No ^a^	No ^a^	Yes	N/A

Adapted from Somer (1993) and D'Arrigo *et al.* (2005) .

^a^ Both children were scanned within the first year of life, when myelination is incomplete and it cannot be assessed if these children were going to have a reduction in cerebral white matter.

All affected children presented at birth with microcephaly [Occipito-frontal head circumference (OFHC) of − 3 standard deviation (SD) to − 4 SD, typically <29 cm] and moderately severe hypotonia. Weight and length at birth were normal. Excepting the microcephaly, they were not dysmorphic, nor did they have any extracranial congenital anomalies. All spent 2–4 weeks in hospital until satisfactory breathing and feeding were established. All developed seizures associated with anoxia presenting at birth or by 1 month. In all three subjects this progressed to flexion infantile spasms with hypsarrhythmia on EEG within the first year of life. Seizures became tonic/clonic by the third year, fits occurred frequently (at least weekly throughout life), status epilepticus necessitating hospital admission at least once in all three, and their seizures were persistently difficult to control necessitating frequent changes of medication and dosage. Over the first 6 months poor visual attention, fixing and following were noted, and all were diagnosed as having severe cortical visual inattention. All had moderate optic atrophy but otherwise a normal retina on fundoscopy; one had minor bilateral megalocornea (10 mm at 6 months, but there were no associated problems from this, such as glaucoma). Puffiness of the maxillary region of the face, and the dorsum of the hands and feet was present from birth, and persisted, but did not spread in extent ( Fig. 1 ). Over the first 3 years of life it became clear that all had profound cognitive delay. Severe motor delay was also present with no development, with a combination of central hypotonia and peripheral hypertonia. Thoraco-lumbar kyphoscoliosis (with no vertebral anomalies) and hip flexion deformity developed by 3 years in two (the third not being old enough). The degree of microcephaly gradually increased over the years of follow up to − 5 SD to − 6 SD by 3 years. MRI was performed on all three affected children and showed the consistent and symmetrical findings shown in Fig. 1 : widespread coarse pachygyria with the posterior regions of the brain more affected than anterior, polymicrogyria prominent in the sylvian fissures, dilated ventricles (reflecting brain substance loss and not raised intraventricular pressure), hypoplastic corpus callosum, subependymal cysts, and hypoplastic pons. One of the three affected children had a small cerebellum shown on an MRI scan at 3 months.

The two families were related, and each set of parents were first cousins ( Fig. 2 ). The parents were healthy, and of normal cognitive and physical abilities, and had no abnormal neurological features. The family were ethnic white Caucasians. Each of the two families also had normal children. In one family a deceased daughter had complete sex reversal and absent adrenal glands, but no PEHO features. No unusual events occurred in the pregnancy or delivery of the affected children, nor was there any exposure to potential teratogens during the pregnancies. We concluded that the condition was inherited as an autosomal recessive trait. The affected children in this family fulfilled the diagnostic criteria for PEHO syndrome; however, the presence of structural brain anomalies (polymicrogyria nor pachygyria) were not described in the original Finnish cases, and so we considered that the better phenotypic diagnosis for the family was PEHO-like syndrome.

### Identification of *CCDC88A* mutation

To identify the causative gene mutation in this family, exome analysis was performed on genomic DNA from two affected children (Patients 1 and 2, Fig. 2 A), the raw sequences mapped to the GRCh37 reference human genome, and any possible mutations shared in both children analysed for their potential to cause PEHO syndrome. We prioritized the search for mutations that would potentially alter the encoded protein (nonsense/frameshift/splice site/missense mutations), and to those that were both homozygous and within concordant homozygous segments shared between the two cousins. Only one deleterious mutation was identified, which was present in both. This was a homozygous frameshift mutation in the *CCDC88A* gene (c.2313delT) creating a TAG stop codon at the site of the frameshift (p.Leu772X) ( Fig. 2 B). Segregation of the mutation was confirmed by Sanger sequencing as homozygous in all three affected children and heterozygous in all of their parents ( Fig. 2 C). The mutation was not identified in the 1000 Genomes Project or ExAC database, and we did not find it in > 200 ethnically matched control chromosomes tested by Sanger sequencing.

*CCDC88A* encodes the actin binding protein girdin, which has previously been demonstrated as a binding partner of Akt and to play essential roles in the migration of fibroblasts, endothelial cells, cancer cells and neuronal cells ( Enomoto *et al.* , 2005 , 2009 ; Jiang *et al.* , 2008 ; Kitamura *et al.* , 2008 ). Mice with germline deletion of girdin exhibit neuronal migration defects which result in hypoplasia of the olfactory bulb, granule cell dispersion in the dentate gyrus and postnatal lethality, demonstrating a crucial role for girdin in the early development of the mouse brain ( Asai *et al.* , 2012 ).

The girdin protein consists of a conserved N terminal domain showing significant homology to the microtubule binding region of the Hook protein family and proposed dimerization domain ( Enomoto *et al.* , 2006 ), a coiled coil central domain, known to bind members of the Gα family of heterotrimeric G proteins ( Le-Niculescu *et al.* , 2005 ) and a C-terminal domain that specifies binding partners including actin and Akt ( Fig. 2 B) ( Enomoto *et al.* , 2006 ). We hypothesized that the introduction of a premature stop codon early within the coiled coil domain would likely result in nonsense mediated decay and hence complete loss of the translated product. However, we were unexpectedly able to identify a reduced amount of full length mRNA, carrying the p.Leu772X in the lymphocytes of Patient 1 ( Fig. 2 D and Supplementary Fig. 1 ), and were able to exclude the possibility of abnormal splicing of the exon containing the c.2313delT mutation ( Supplementary Fig. 1 ). While it is highly likely that this mutation would nonetheless produce a complete knockout of the girdin protein, mRNA extracted from the brains of a *Ccdc88a* knockout mouse produced by excising exon 3 to produce a premature stop codon also displayed presence of the mutated *Ccdc88a* transcript ( Asai *et al.* , 2012 ), we proceeded to confirm the pathogenicity of the truncated girdin by cloning it into HEK293 cells as a haemagglutinin-tagged fusion protein. Wild-type girdin produced a single band at ∼250 kDa by western blot, whereas introduction of the c.2313delT mutation by site-directed mutagenesis resulted in a truncated product of around 105 kDa in size ( Fig. 2 E). Many of girdin’s reported functions are reliant on its interaction with C-terminal binding partners (including actin, Par-3, Akt, multiple receptor tyrosine kinases and Gαi3; Enomoto *et al.* , 2005 , 2006 ; Ohara *et al.* , 2012 ; Lin *et al.* , 2014 ; Bhandari *et al.* , 2015 ) and its guanine nucleotide exchange factor domain, membrane binding region and a number of functional phosphorylatable residues lie downstream of the premature stop codon introduced ( Bhandari *et al.* , 2015 ). It seems probable that, in the unlikely event that the truncated protein is produced *in vivo* , the function of the mutant protein would be highly disrupted due to its absent C terminal functions.

### Features of PEHO syndrome in *Ccdc88a* knockout mice

Having proven the potential pathogenicity of the p.Leu772X mutation in cellular models we asked if the knockout of *CCDC88A* in the mouse might mimic features of PEHO syndrome and could be used to identify molecular defects in the mouse brain responsible for the PEHO phenotype, which are unable to be investigated in affected humans. Mice with a knockout of *CCDC88A* have previously been reported to show a mesial-temporal lobe epilepsy phenotype, and this was again found ( Asai *et al.* , 2012 ). The mice also show postnatal lethality and postnatal growth retardation being reported underweight at death ( Asai *et al.* , 2012 ). This phenotype is generally consistent with that of PEHO syndrome.

**Figure 3:**
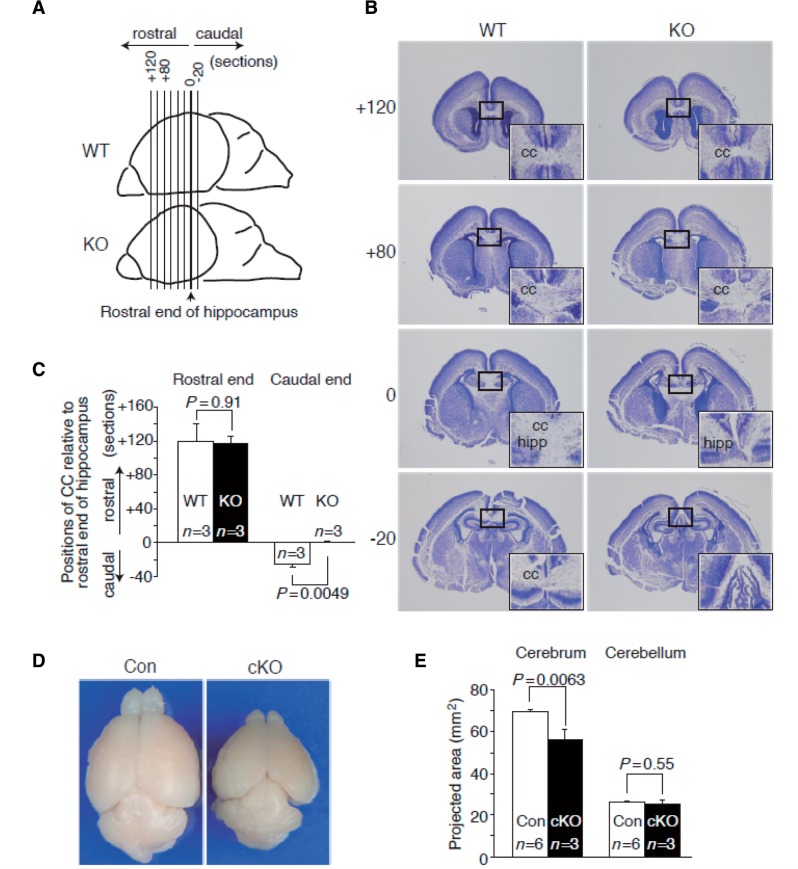
***Ccdc88a* -deficient neonatal mice (postnatal Day 0) exhibit mild hypoplastic defect in the caudal end of corpus callosum at midline.** ( **A** ) Paraffin embedded brain tissues from wild-type mice (WT) or straight *Ccdc88a* deficient mice (KO) were coronally sectioned at 7-mm thickness. All sections were serially numbered using the rostral end of hippocampus as a reference section. Rostral sections are positively numbered, and caudal sections were negatively numbered. ( **B** ) Nissl stained coronal sections of wild-type and knockout brain. Positions of corpus callosum (CC) relative to the rostral end of hippocampus were similar between wild-type and knockout mice (at around +120 section), while the caudal end of knockout mice corpus callosum was relatively displaced forward compared with the caudal end of wild-type corpus callosum (wild-type at around −20 section level, knockout at around the reference section). hipp = rostral end of hippocampus. Black square indicates the area of each inset at bottom right corner. ( **C** ) Statistical analysis of positions of corpus callosum relative to the rostral end of hippocampus. Three replicates from each genotype were analysed. Section numbers of the rostral end of corpus callosum from the reference section were averaged. In accordance with the observations in **B** , the caudal ends of corpus callosum of knockout were significantly displaced to rostral side. Values are shown as mean plus standard error. T-test P values less than 0.05 were considered significant. ( **D** ) Overhead view of *Ccdc88a* wild-type/flox:nestin-Cre(−) control mouse (Con) and *Ccdc88a* flox/flox:nes-Cre(+) conditional knockout mouse (cKO). ( **E** ) Horizontal projected area was measured and analysed. Cerebrum of cKO was significantly smaller than control mice, while areas of cerebellum were not different between controls and cKO mice.

We proceeded to assess the brains of *Ccdc88a* deficient mice at an anatomical level. Completeness of corpus callosum development in *Ccdc88a* deficient mice was assessed with Nissl stained coronal sections of neonatal (postnatal Day 0) brain tissue. There was no global difference in appearance of corpus callosum between wild-type and *Ccdc88a* deficient mouse, while there was a significant developmental defect in the caudal end of corpus callosum in knockout mice ( Fig. 3 A–C). This may match the finding of a hypoplastic corpus callosum observed in *CCDC88A* mutant human patients. Layer structures of cerebrum at postnatal Day 12 were compared between wild-type and knockout using Kluver-Barrera staining and immunohistochemistry with layer specific markers (Cux1 for layer II–III, and Ctip2 for layer V). With Kluver-Barrera staining, no ‘polymicrocyria’ like lesions were observed. Immunohistochemistry with layer markers didn’t detect any abnormal layer structure, except that staining intensity of Ctip2 was slightly less in knockout mice. To assess optic nerve atrophy, the cross-section area of optic nerves were measured with ImageJ (National Institute of Mental Health, Bethesda, MD), and averaged. There was no significant difference between wild-type and knockout mice (wild-type versus knockout, *n* = 6 versus 5, mean 0.0241017 versus 0.025981 mm ^2^ , SD 0.003509 versus 0.003313 mm ^2^ , *t* -test *P* -value 0.384). To assess microcephaly, analysis of overhead view pictures of brains from nestin-Cre driven *Ccdc88a*^flox/flox^ mice (cKO) at postnatal Day21 were carried out using ImageJ. Projected area of cerebrum of cKO was significantly smaller than that of *Ccdc88a* wild-type/flox nestin-Cre (-) (control), while areas of cerebellum of cKO and control were similar (Fig. 3D and E ).

## Discussion

We investigated a multi-affected consanguineous family of British origin with three children born with features of PEHO syndrome. By comparing the exomes of consanguineous first cousins we detected a single nucleotide deletion in exon 15 of *CCDC88A* (c.2313delT). This results in a frameshift and incorporation of a stop codon (p.Leu772X). Our results confirm an autosomal recessive pattern of inheritance for PEHO syndrome, and identify *CCDC88A* as a further gene essential for normal human brain development.

*CCDC88A* encodes the actin binding protein girdin, a multi-domain, non-receptor guanine exchange factor (GEF) for G proteins Gαi1, Gα2 and Gα3, which has been attributed a wide range of functions including roles in cell migration and Akt/EGFR signalling ( Enomoto *et al.* , 2006 ; Beas *et al.* , 2012 ; Bhandari *et al.* , 2015 ), actin organization and cell motility ( Enomoto *et al.* , 2005 ), neurite outgrowth and migration, axon/dendrite development ( Enomoto *et al.* , 2009 ; Wang *et al.* , 2011 ) and to regulate early endosome maturation ( Beas *et al.* , 2012 ). Girdin can link actin filaments through its C terminal domain and simultaneously bind the plasma membrane through the adjacent membrane binding domain at the protein C terminus. This allows girdin to function in actin cytoskeletal remodelling during cell mobility ( Hu *et al.* , 2015 ). Other genes reported to cause cortical lamination defects (lissencephaly, pachygyria and polymicrogryia), severe developmental delay and seizures, are also generally cytoskeletal proteins (e.g. the tubulins) or act upon cytoskeletal proteins [LIS1 ( *PAFAH1B1* ), DCX, reelin ( *RELN* ), NUDE ( *NDE1* ) and NDEL1], and similar to *CCDC88A* , have complex multiple interactions, and functions ( Guerrini and Dobyns, 2014 ).

Postnatally, girdin is expressed in the majority of mammalian tissues; however, during embryonic development, the expression pattern of CCDC88A is specifically restricted, both spatially and temporally to regions of the fore-, mid- and hindbrain as well as the dorsal root ganglia, somites, limbs, nasal processes and branchial arches ( Simpson *et al.* , 2005 ). This specific expression profile implies a potentially important role during embryonic development.

The identification of a truncating mutation in *CCDC88A* in individuals with PEHO syndrome confirms a crucial function for girdin during embryonic development. Patients with a mutation in *CCDC88A* resulting in either a truncated girdin or absence of the protein due to nonsense mediated decay, displayed all of the necessary clinical criteria to be diagnosed with PEHO syndrome. All presented at birth with microcephaly and moderately severe hypotonia and developed seizures that progressed into infantile spasms with hypsarrhythmia on EEG. All also displayed progressive brain atrophy, optic atrophy, severe cognitive delay and puffiness of the maxillary region of the face, and the dorsum of the hands and feet. Reassessment of knockout mice for *Ccdc88a* demonstrated further confirmation of *CCDC88A* as a PEHO-causing gene, with mice displaying a similar phenotype with mesial-temporal lobe epilepsy and early demise, and structural brain developmental defects affecting the corpus callosum and cerebrum. A lack of macroscopic pachygyria was expected as the murine cerebral cortex is ‘smooth’ and without a gyral pattern. However, the finding of only a minor (layer 5) cortical lamination defects in *Ccdc88a* mice may have been unexpected; the human/mouse correlations for other similar brain–cytoskeletal genes are variable and unexplained: e.g. *DCX* mutations cause pachygyria in humans and the knockout mouse shows only hippocampal lamination defects ( des Portes *et al.* , 1998 ; Corbo *et al.* , 2002 ); *NDE1* mutations cause lissencephaly in humans and the knockout mouse has microcephaly, but cortical lamination is mostly preserved ( Feng and Walsh, 2004 ; Wynshaw-Boris *et al.* , 2010 ).

Recently, an individual with PEHO syndrome was reported who harboured a *de novo* missense mutations in *KIF1A* , and the authors noted up to six similar previously reported cases ( Langlois *et al.* , 2015 ). Of other individuals with significant mental handicap and *KIF1A* missense mutations, most had prominent and progressive spasticity and neuropathy—findings atypical of PEHO syndrome ( Okamoto *et al.* , 2014 ; Esmaeeli Nieh *et al.* , 2015 ; Lee *et al.* , 2015 ; Ohba *et al.* , 2015 ). Furthermore, *KIF1A* mutations have also been reported to cause dominant and recessive pure spastic paraplegia, recessive sensory neuropathy and dominant mental retardation (OMIM: 601255). Thus, the PEHO syndrome phenotype can also have a dominant, and not just a recessive, inheritance pattern caused by a subset of *KIF1A* missense mutations.

To conclude, we report the first identification of a gene, *CCDC88A* , which when mutated causes an autosomal recessive PEHO-like neurodevelopmental phenotype. We detail the human phenotype caused by the lack of functioning GIRDIN, and found similar neurological features and anatomy in *CCDC88A* knockout mouse.

## Funding

This work was supported by the MRC and Cambridge University Hospitals NHS Foundation Trust (M.S.N.). Y.C.C. and C.G.W. are supported by the Cambridge NIHR Biomedical Research Centre. This work was also supported by A-STEP from the Japan Science and Technology Agency in 2014 (AS251Z02522Q) and in 2015 (AS262Z00715Q), a Takeda Visionary Research Grant 2014 from the Takeda Science Foundation, a Grant-in-Aid for Scientific Research (C) (to M.A.), and a Grant-in-Aid for Scientific Research (S) (to M.T.) from the Ministry of Education, Culture, Sports, Science, and Technology in Japan.

## Supplementary material

Supplementary material is available at *Brain* online.

## Supplementary Material

Supplementary Data

## Abbreviation

PEHO: progressive encephalopathy with oedema, hypsarrhythmia and optic atrophy
