# Supplementary material for: CCDC88A mutations cause PEHO-like syndrome in humans and mouse
Source: Brain. 2016 Feb 25;139(4):1036–44. doi: 10.1093/brain/aww014 (PMC4806221; doi:10.1093/brain/aww014)
Supplement: Supplementary Data [file aww014_supplementary_data.zip › Supplementary_Material.pdf]

Supplementary Materials

MATERIALS AND METHODS

Subjects

The UK National Research Ethics Service, Cambridge Central Research Ethics committee gave permission for our study. The authors cared for the family for over a decade; all medical care was within the National Health Service, results and notes were examined.

Exome Sequencing and Mutational analysis

Using standard methodologies genomic DNA was isolated from blood and stored from all three living affected children and their parents. Exome sequencing was performed on two affected children (children 1 and 2 in Figure 2a) using the SureSelect Human All Exon 50Mb Kit (Agilent Technologies UK, Cat.No G3370A) according to manufacturer's instructions, producing targeted capture of 4Mb sequence. Sequencing was performed with the SOLiD^{TM}4 system (Applied Biosystems, UK) to generate an average of 1.2 Gb of sequence. The average read depth was 181, and 88.3% of exons had a coverage of >9 reads (sufficient for identification of homozygous mutations). The raw sequencing reads were then mapped to the GRCh37 reference human genome and changes compared to the reference sequence. In trying to identify a causative mutation, we filtered based upon changes not present in UTR's or intronic regions, or at <1 in 500 in the 1000 Genomes project, and focussed on homozygous changes shared in both affected siblings. Changes in nonsense mediated decayed transcripts were dismissed. Any changes shared were confirmed using the Integrated Genome Viewer and then potential mutations sequenced by Sanger sequencing in the affected children. The segregation of the *CCDC88A* mutation identified within the family was assessed by polymerase chain reaction and Sanger sequencing (primers F:

AGAACTGCGAAGGAATGTAGA R: TCAATAGTTGCTCTTTTCACAAG). Affected child 1 and 2 shared a 18.48Mb concordant homozygous region centred upon *CCDC88A*.

Reverse transcription PCR analysis

Separate blood samples were taken from child 1 and their parents and RNA extracted. Semi-quantitative RT-PCR was carried out to determine if patient cDNA harbouring p.Leu772X was detectable in mRNA extracted from affected child 1, and secondly if there was any evidence that exon 15, encompassing the mutation, was spliced out of the mature mRNA. Quantitative Real Time RT-PCR was undertaken to detect if there was a reduction in level of the mutant transcript indicating evidence of nonsense mediated decay.

To extract mRNA from the lymphocytes from affected child 1 and his unaffected parents, the QIAamp RNA blood mini kit (Qiagen) was used and the mRNA converted to cDNA using the SuperScript® III Reverse Transcriptase (Life Technologies,). Exon spanning primers to the 5' end of the mutation site (used for qPCR and RT PCR) were F: GCCTCACTTAGAATGCACAATCT R: ACTTCCATCCATTGCAGGTCA (exon 3–7). Primers from exon 13 to exon 16 to check for possible splicing out of mutant exon were F:GAGCTTCGGACTACTGTGGA R:GGGTCTTCAACTTTTCACTCACC. Primers for the 3' end RT PCR were F:GACGGACAACTAAGCCTGAGT R:TTGTTGCTCCCTAGACCTGC. Quantitative RT PCR (qPCR) was carried out using SYBR select master mix (Life Technologies) and was run using a 7900HT fast Real Time PCR system (Applied Biosystems) according to the manufacturer's protocol.

CCDC88A cloning and expression

CCDC88A cloned into the pCAGGS vector has been described previously (Enomoto *et al.*, 2005). The c.2313delT mutation was introduced into the WT *CCDC88A* sequence by site-

directed mutagenesis using the QuikChange II Site-Directed Mutagenesis kit (Agilent), and the sequence of the mutated plasmid confirmed by Sanger sequencing. A HA tag was added to the N-terminal of both pCAGGS Girdin WT and pCAGGS Girdin 2315delT using primers: GCAGCTCGAGCGCCACCATGTACCCATACGATGTTCCAGATTACGCTATGGAGA ACGAAATTTTTACTCCCCTTCT and GCAGGATATCTGTTCCAGTTCCCAGCCAAG.

Presence of Girdin was confirmed by western blot, using purified anti-HA.11 antibody (Biolegend).

<u>CCDC88A Knockout mouse</u>

Both the *CCDC88A* knock out mice and conditional Nestin cre knockout mice have been reported previously (Kitamura *et al.*, 2008, Asai *et al.*, 2012). Wild type (WT) or ccdc88a deficient mice (KO) neonates (P0) were obtained by mating of heterozygous parents. Neonates were transcardially perfused with 10% neutral buffered formalin (Muto Pure Chemical, Tokyo, Japan). Dissected brain tissues were post-fixed in 10% neutral buffered formalin (Muto), and dehydrated in increasing concentrations of ethanol. Immersion solutions were then displaced with xylene, and molten paraffin. Paraffin block was sectioned at 7 □m thickness. All sections were mounted on MAS coated glass-slides (Matsunami glass, Osaka, Japan), deparaffined, stained with cresyl violet acetate (Merck, Darmstadt, Germany), and photographed with a microscope (Olympus, BX53, Tokyo, Japan) using imaging software CellSens standard (Olympus).

<u>Clinical Description of Affected PEHO cases</u>

The first affected child ascertained is indicated as “1” in Figure 2 and was born after an uneventful pregnancy and labour. He presented at birth with hypotonia and borderline microcephaly, a sloping forehead and micrognathia. His occipital frontal head circumference at birth was 31.8cm (0.4th centile) and 37cm at 4 months (<0.4th centile), which progressed to

occipital frontal head circumference of < -3SD by a year. He started to have seizures in the first week of life, these evolved into infantile spasms, and he continued to have seizures which were extremely difficult to control throughout his life; EEGs showed hypsarrhythmia. He had puffy cheeks and backs of his hands and feet and was considered to have the appearances of PEHO syndrome (see Figure 1). Nuclear magnetic resonance brain imaging at four years showed: a simplified gyral pattern; posterior and occipital lobe irregular thickening which was suspected to be polymicrogyria; a reduced amount of white matter; a thin corpus callosum; and pontine hypoplasia. At 11 years of age he is profoundly cognitively and physically handicapped, has made no developmental progress, has intractable seizures, and behaves as if almost blind and had optic atrophy. On examination he has microcephaly (OFC 42.3 at 2.5 years), severe spasticity with dislocated "windswept" hips, scoliosis, and bilateral undescended testes. The family had lost their first child, a daughter, with complete sex reversal and absent adrenal glands – the aetiology of which is unknown.

The second family had two affected children, a boy "2" and a girl "3" in Figure 2. At birth the boy was noted to be hypotonic, microcephalic (occipito-frontal head circumference 32cm [0.4th centile]) and had mild growth retardation (height and weight on 3rd centile for age and sex). He began having seizures on his first day. At seven months his seizures were reported to occur at least 4-5 times a day where he would shake for a few seconds and then the arms and legs would become stiff. Several episodes would occur at short intervals both day and night and typically he would have 3-4 seizures before sleeping. During the first year he developed the facial appearance of PEHO syndrome and had mild but persisting swelling of the dorsum of his hands and feet. During the first year it became clear that he had significant developmental delay, severe visual impairment and secondary microcephaly. Magnetic resonance imaging at three months showed polymicrogyria, considered consistent with a

diagnosis of Type 1 Lissencephaly, dilated lateral ventricles, subependymal cysts and a hypoplastic pons (Barkovich *et al.*, 2012). From the end of the first year onwards he progressively developed spastic quadriplegia. Seizures continued, included infantile spasms with an EEG showing hypsarrythmia and were difficult to control requiring multiple anti-epileptics. His cognitive abilities were limited to recognising his parents, responding to pain and indicating he wanted feeding. He was unable to communicate anything more than basic needs. He was functioning at the level of a baby of 0-1 months, but he lost no skills. His condition was considered developmental and static but not progressive nor degenerative.

Following the birth of the family's first affected child their next pregnancy was carefully monitored. It was a twin pregnancy of a male and female; the female was affected and the male un-affected. Prenatal ultrasound scanning showed ventriculomegaly in the female from 20 weeks of gestation but all other measurements and anatomy was normal. On follow up over the remainder of the pregnancy the ventriculomegaly gradually resolved but the growth of the occipito-frontal head circumference diminished in the later third trimester. No structural brain anomalies were detectable by ultrasound prior to the third trimester. At birth she was microcephalic (occipito-frontal head circumference 28.8cm (<0.4th centile), but with height and weight within the normal range), and hypotonic, and within three hours of birth she developed short self-limiting seizures. Because of this she was ventilated for the first five days of life. She also developed the facial appearance of PEHO syndrome and had persisting swelling of the dorsum of his hands and feet within the first three months. As with her older affected brother, central hypotonia persisted and she developed a spastic paraplegia. Her seizures continued to be frequent to be difficult to control, lasting between 10-40 seconds occurring about 10 times per day in total. She too had visual inattention which was considered primarily cortical in origin, although she did have minor bilateral optic atrophy.

Her cognitive abilities were severely restricted and she has made little developmental progress. Her magnetic resonance brain imaging was performed at three months without movement artefact and on a machine capable of a higher resolution image than achieved with the other affected family members. It showed: symmetric pachygyria and polymicrogyria with shallow sulcations over the frontal, temporal and occipital regions; coarse polymicrogyria less evident anteriorly than posteriorly; hyperplastic and incompletely formed corpus callosum; hypoplastic pons; and a hypoplastic cerebellum of normal architecture.

The affected children were extensively investigated and had collectively these normal investigations: urine organic and amino acids, blood renal and hepatic function tests, amino acids, lactate, vitamin B12, transferrin isoelectric focussing, lysosomal storage enzymes, creatinine kinase and thyroid function tests including T4; chromosome analysis by cytogenetic microscopy and microarray, TORCH screens for prenatal infection, full blood count and film, cerebrospinal fluid protein, pterins pyridoxal phosphate, 5-methyltetrahydrofolate, renal, bladder and heart ultrasound scans. *LIS1* and *ARX* mutations were excluded by clinical-grade Sanger sequencing.

<u>Inconclusive evidence for nonsense mediated decay of p.Leu772X transcript</u>

In order to test for possible nonsense mediated decay of mRNA harbouring the p.Leu772X mutation, reverse transcription PCR analysis was carried out on cDNA extracted from the lymphocytes of affected child 1 and his unaffected parents. Exon spanning primers were designed to the 5' end of the p.Leu772X mutant transcript, and the PCR reaction optimised and visualised. Unexpectedly a band was identified in the cDNA extracted from the affected child (Figure 2D). We confirmed that exon 15 containing the mutation was not spliced out by

designing primers encompassing this region and confirmed by Sanger sequencing that this band did include the homozygous p.Leu772X mutation (see Supplementary figure S1A). Both parent's cDNA was also confirmed to be heterozygous for the mutation. We also ran an RT PCR amplifying a region 3' of the c.2313delT mutation (see Supplementary figure S1B). Finally we undertook SYBR green quantitative RT-PCR (Applied Biosystems) using the original 5' primer set and optimised the reaction for specific amplification of the cDNA product. We identified reduced mRNA levels in the affected child compared to the unaffected parents and an unaffected control, but not complete loss of the patients mRNA (see Supplementary Figure S1C).

Reference

Barkovich AJ, Guerrini R, Kuzniecky RI, Jackson GD, Dobyns WB. A developmental and genetic classification for malformations of cortical development: update 2012. Brain 2012; 135: 1348–69.

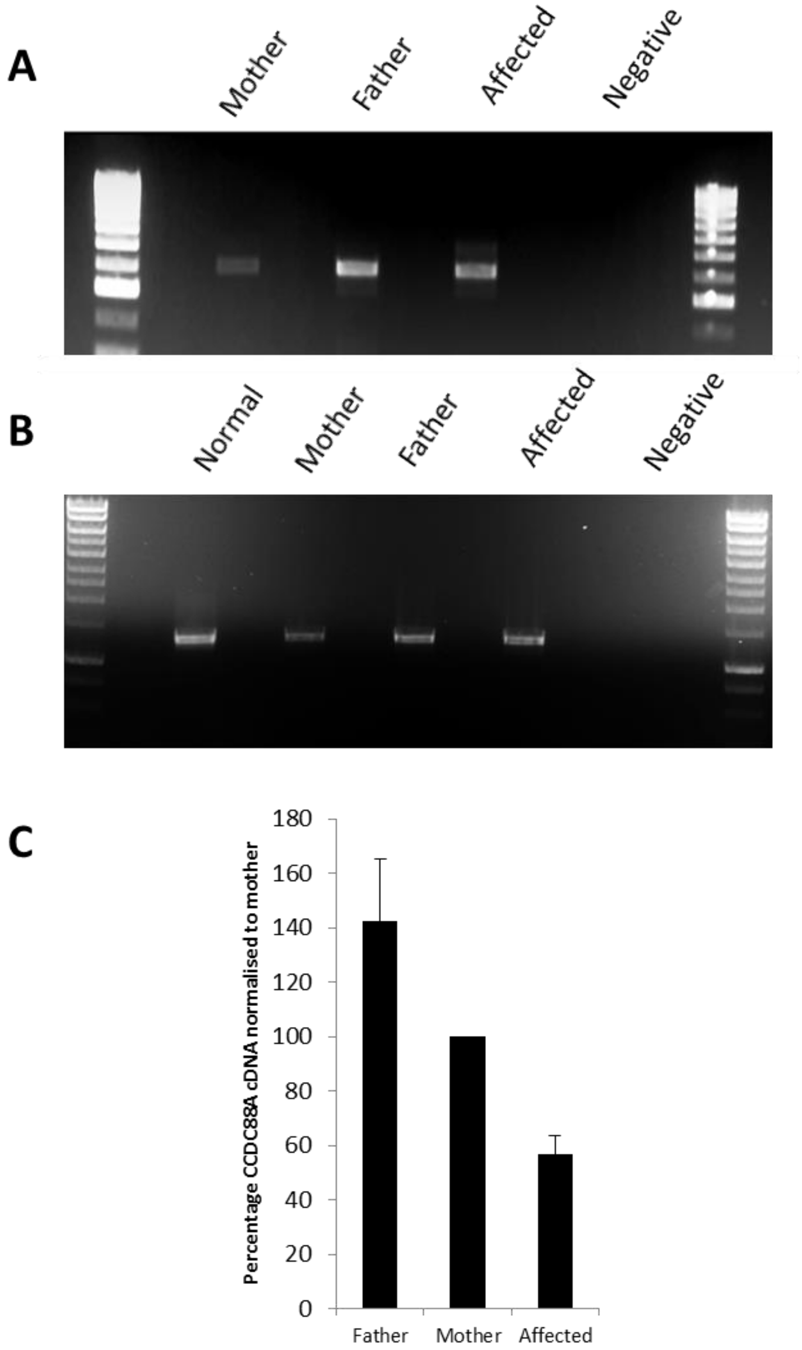

<u>**Supplement Figure S1: Inconclusive evidence of complete nonsense mediated decay of *CCDC88A* in PEHO affected child**</u>

A: RT-PCR confirming the presence of the mutation containing exon 15 in patient cDNA (this PCR was also sequenced to confirm presence of the mutation).

B: RT-PCR confirming the presence of cDNA transcript 3’ to the frameshift mutation

C: Quantitative SYBR green RT-PCR suggests a reduction but not total loss of patient cDNA compared with unaffected parents.
