# Supplementary figures and images for: CCDC88A mutations cause PEHO-like syndrome in humans and mouse
Source: Brain. 2016 Feb 25;139(4):1036–44. doi: 10.1093/brain/aww014 (PMC4806221; doi:10.1093/brain/aww014)

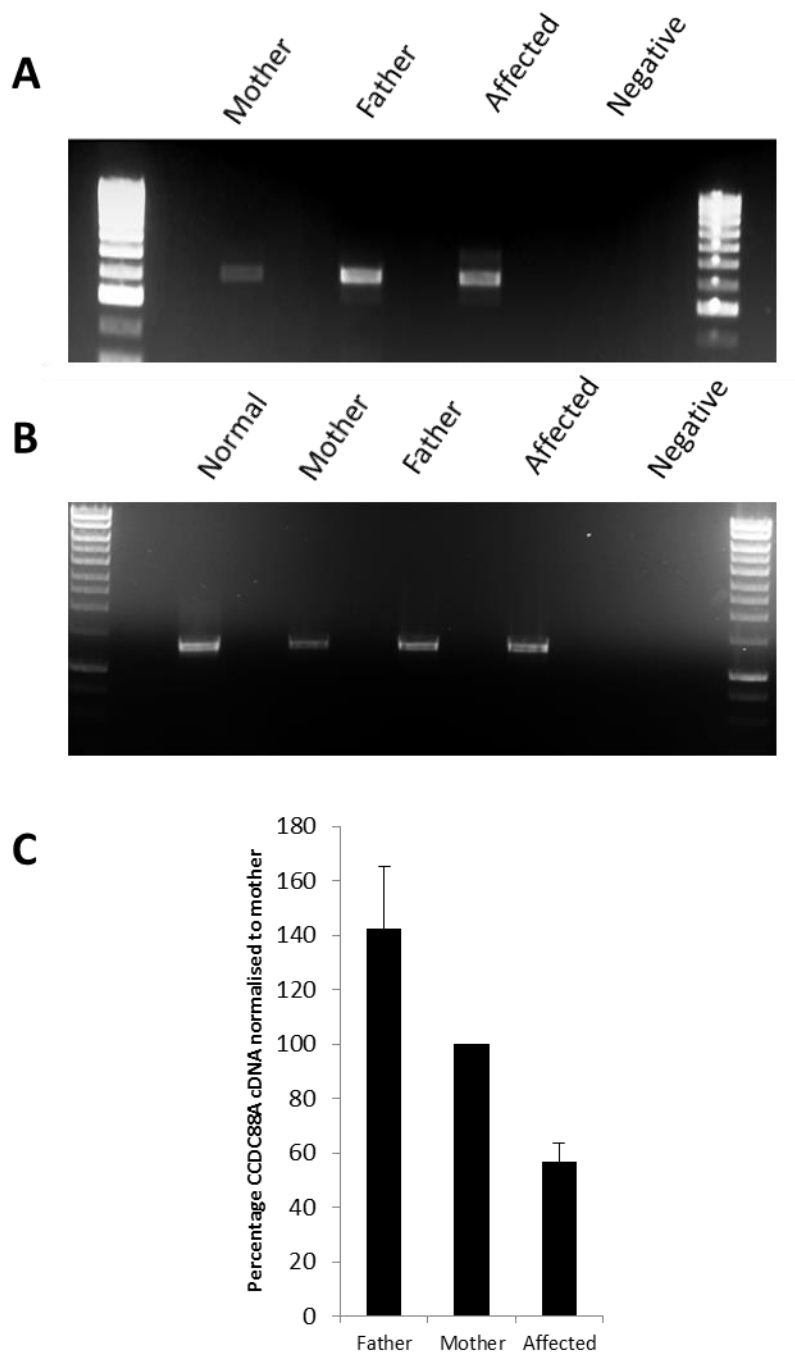

Inconclusive evidence of complete nonsense mediated decay of CCDC88A in PEHO affected child

Supplement: Supplementary Data [file aww014_supplementary_data.zip › Supplementary_Figure_1.pdf]
